# A Trip through Southeast Asian Airports in Times of COVID-19

**DOI:** 10.4269/ajtmh.20-0153

**Published:** 2020-03-17

**Authors:** Lorenz Vock

**Affiliations:** Karl Landsteiner University of Health Sciences, Department of Opthalmology, Dunant Platz 1, 3100 St Poelten, Austria

I am just an ophthalmologist. I have no special training in hygiene or tropical medicine, and I am not reporting on a special case or treatment but on a layman’s observation on hygiene in airports. As anybody going on holiday to Southeast Asia, I just took the usual precautions: antibiotics in case of diarrhea, disinfectants, masks, etc. Nothing was known about the 2019 novel coronavirus disease (COVID-19) at that time when the journey was planned. Time went on, and then came the COVID-19 outbreak in China. People were astonished and curiously watching hospitals being erected in China within 2 weeks on TV. But here in Europe, people were relaxed. It was all far away.

Fear only grew as more and more news about the outbreak of COVID-19 reached the world. People were canceling their holidays not only to China but also to other countries. Warnings concerning economic growth were issued worldwide as the virus spread. Suddenly, people became aware how vulnerable they would be in a world where infectious particles could spread in 24 hours by plane.

The less it was known about the disease, the more fantastic the stories spread. For example, many of the Cambodians I met believed that the virus could not survive more than 30°C. This was why they were safe, whereas the Chinese were in danger in Wuhan because the climate there would be much colder. But why would our authorities calm the public that the disease was not that dangerous, while one could see quarantine and other measurements being taken up at the same time and while the daily death toll in China and other countries kept rising?

Anyway, we decided not to cancel the trip. Coming from Europe, we had no special treatment to expect when landing in Bangkok. All passengers had to pass an infrared camera to single out febrile passengers. But the camera was not positioned at the gate where we disembarked, but at the junction of several terminals where passengers form all over the world were passing by to find their way either to the exit or to a connecting flight. We passed the infrared camera very fast in a single line. Some people were wearing facial masks—nothing uncommon for Asia. No questions were asked. Nobody was stopped. Anyway, the shear mass of passengers passing by would have clogged up the capacities of the controlling personnel in my view.

What struck me more was the frequent control of documents. Personal security and terrorism obviously played a bigger role in the authorities’ minds than health. I counted four different passport and boarding pass checks, where my documents were physically handled before the connecting flight. All of the security personnel took my passport and boarding pass, as well as those of hundreds of other passengers, in their bare hands. There seemed to be a strong need for a haptic control of documents.

I felt a bit uneasy. It was reported at that time that China was collecting and disinfecting old banknotes to prevent the spread of COVID-19. The mode of transmission of the disease still was partly in the dark: could there be smear infection as well? When I asked to be allowed to use one of the few disinfectant dispensers, a friendly officer pushed the pump on the dispenser with her bare hands.

Finally, we made the connecting flight. Several times it was announced that the flight attendants were required by their airline to wear facial masks to inhibit the spread of the virus. They only had very simple masks, just good enough to avoid a direct hit of big droplets if somebody sneezed. As soon as the attendants were seated themselves, they took the masks off and put them on again when serving the passengers. I thought about what I knew about air conditioning in planes. Not very much. Do they use fresh air or is air kept in circulation? What filters do they use?

Landing in Siem Reap was no different. The procedure of obtaining a visa on arrival was like a flashback to past times. Passengers were lining up in front of a huge desk with uniformed officers looking fierce. There were about 10 in a line. The procedure was simple. You handed in your passport and paperwork to the first officer and paid your dues. Then, you advanced along the desk at the same time as your passport was handed from one officer to the other being processed. The lack of machinery made this process a quite efficient and fast way to issue the visa—and spread particles. And yes, you would guess right that documents were manipulated by bare hands and there was no disinfection of said hands to be noticed.

Cambodia is a beautiful country with a wealth of cultural sights. I had it mostly for myself as tourists were rare at the time when the Westerdam crisis occurred. You might remember, the Westerdam was a cruise liner suspected to carry passengers being infected with the SARS-CoV-2. It was turned away from several countries before being allowed to dock in Cambodia. The passengers were allowed to leave and apparently had insufficient medical screening before being allowed to go on their way. As a result, Thai authorities raised vigilance for arrivals from Cambodia. I already thought that I was going to get stuck in the Bangkok airport on my way back.

But none of that happened. I thought that health controls would now take place right at the gate of the docking plane. But I was wrong. Again, we passed an infrared camera at the juncture of several terminals. Multiple times my passport and boarding pass were checked in the manner described earlier, but this time I had to give fingerprints at the immigration checkpoint. Laser scanners were used to this purpose, and I had to put all fingers and thumbs on a glass screen to be scanned.

The glass screens were full of fingerprints of previous passengers and even remnants of food, I guessed. A sign told passengers that the fingerprint screens were cleaned every hour. I was trying to figure out how many passengers would put their fingers on such a fingerprint scanner within 1 hour when I was suddenly stopped at the exit of the control unit.

It was now that the authorities became aware that I was arriving from Cambodia after having traversed half of the airport. I was asked to wait separately with two or three other passengers having the same fate. My passport was once again scrutinized manually by several officers. There was excitement, and it seemed that they did not quite know what to do with me, and while there was some discussion among the officers, other passengers slipped through the controls simply because there was nobody at the desks.

Finally, after some time, an officer returned to me with my passport, handed it over, and asked me whether or not I had passed the infrared camera. She was very polite and apologized for the inconvenience. When I confirmed, I was free to go.

Now, why would one tell such a story, which is—after all—not extraordinary for the readers of this journal. It is certainly not to blame authorities at airports. This could be happening at any airport anywhere in the world.

This trip made me think that humankind is simply not prepared for the COVID-19 outbreak. The next virus or infectious particle is sure to come. It might be much worse. In addition to the need for scientific progress in this field, we also have to change some of our habits, and we need to educate. Precaution, understanding, and hygienic habits must become the rule for everybody. Otherwise, quarantine and other measures of enforcement will not be sufficient to limit future outbreaks.

